# Host Adaptation of *Chlamydia pecorum* towards Low Virulence Evident in Co-Evolution of the *ompA*, *incA,* and ORF663 Loci

**DOI:** 10.1371/journal.pone.0103615

**Published:** 2014-08-01

**Authors:** Khalil Yousef Mohamad, Bernhard Kaltenboeck, Kh. Shamsur Rahman, Simone Magnino, Konrad Sachse, Annie Rodolakis

**Affiliations:** 1 INRA, UR1282 Infectiologie Animale et Santé Publique, Nouzilly, France; 2 Department of Pathobiology, College of Veterinary Medicine, Auburn University, Auburn, Alabama, United States of America; 3 Istituto Zooprofilattico Sperimentale della Lombardia e dell’Emilia Romagna “Bruno Ubertini”, National Reference Laboratory for Animal Chlamydioses, Sezione Diagnostica di Pavia, Pavia, Italy; 4 Friedrich-Loeffler-Institut Jena, OIE and National Reference Laboratory for Chlamydiosis, Jena, Germany; University of California, San Francisco, University of California, Berkeley, and the Children’s Hospital Oakland Research Institute, United States of America

## Abstract

*Chlamydia* (*C.*) *pecorum*, an obligate intracellular bacterium, may cause severe diseases in ruminants, swine and koalas, although asymptomatic infections are the norm. Recently, we identified genetic polymorphisms in the *ompA*, *incA* and ORF663 genes that potentially differentiate between high-virulence *C. pecorum* isolates from diseased animals and low-virulence isolates from asymptomatic animals. Here, we expand these findings by including additional ruminant, swine, and koala strains. Coding tandem repeats (CTRs) at the *incA* locus encoded a variable number of repeats of APA or AGA amino acid motifs. Addition of any non-APA/AGA repeat motif, such as APEVPA, APAVPA, APE, or APAPE, associated with low virulence (*P*<10^−4^), as did a high number of amino acids in all *incA* CTRs (*P* = 0.0028). In ORF663, high numbers of 15-mer CTRs correlated with low virulence (*P* = 0.0001). Correction for *ompA* phylogram position in ORF663 and *incA* abolished the correlation between genetic changes and virulence, demonstrating co-evolution of *ompA*, *incA*, and ORF663 towards low virulence. Pairwise divergence of *ompA*, *incA*, and ORF663 among isolates from healthy animals was significantly higher than among strains isolated from diseased animals (*P≤*10^−5^), confirming the longer evolutionary path traversed by low-virulence strains. All three markers combined identified 43 unique strains and 4 pairs of identical strains among all 57 isolates tested, demonstrating the suitability of these markers for epidemiological investigations.

## Background

*Chlamydia* (*C.*) *pecorum*, a Gram-negative obligate intracellular bacterium, is a species of the genus *Chlamydia* belonging to the family *Chlamydiaceae* [1]. *C. pecorum* strains have been isolated worldwide from ruminants and swine with conjunctivitis, encephalomyelitis, enteritis, pneumonia, polyarthritis, abortion, and reproductive or urinary tract diseases [2]–[4]. More recent studies have shown that wild animals may also be infected with *C. pecorum*, most prominently Australian marsupials, such as koalas, in which fertility is severely compromised by urogenital infections [5], and western barred bandicoots with conjunctivitis [6]. *C. pecorum* is also found in the conjunctiva, intestine, and vaginal mucus of clinically healthy ruminants and swine [7]–[9]. In fact, such asymptomatic *C. pecorum* infections are found very frequently, particularly in ruminants at high population density where prevalence rates can approach 100% [10], [11], but also in pigs [12]. While high *C. pecorum* infectious loads associate significantly with disease symptoms [13], the majority of *C. pecorum* infections are asymptomatic and very low infectious loads are detected [8]–[10]. Nevertheless, even such asymptomatic infections of calves cause detectable lung dysfunction [14] and incur substantial reductions in weight gains [15]. Collectively, these observations raise the question if the parallel occurrence of asymptomatic and clinically manifest *C. pecorum* infections associates with virulence differences of strains that can be detected and characterized.

For several decades, serotyping using polyclonal or monoclonal antibodies in micro-immunofluorescence assays was used to characterize and classify individual chlamydial strains. Meanwhile, genotyping based on PCR and sequencing of *ompA* has gradually replaced serotyping. Indeed, several new methods were proposed, such as DNA microarray testing [16], multi-locus sequence typing (MLST) [17] and typing based on variable number tandem repeats (VNTR) [18]. However, none of these methods is congruent with the virulence of chlamydial isolates, although some parameters are correlated with clinical manifestations and serotyping. For *C. trachomatis*, in a study including 175 men and 135 women attending a sexually transmitted disease (STD) clinic, a correlation was reported between urethral discharge in men and serotypes H and J, and between lower abdominal pain in women and serotypes F and G [19]. Furthermore, 47.5% of asymptomatic patients were infected with *C. trachomatis* serovar E among 1,770 STD-infected women in China [20]. As to *C. psittaci*, serovar D strains induce the most severe disease in turkeys [21].

*C. pecorum* strains present many genetic and antigenic variations [22], [23]. In earlier investigations, we found virulence-associated genetic differences among 19 *C. pecorum* strains by identifying different motifs of the variant coding tandem repeats (CTR) in *incA* of isolates from sick versus healthy ruminants [24]. By determining lower numbers of repetitions of the CTR in the hypothetical ORF663 in highly virulent *C. pecorum* strains than in low-virulence isolates, we further identified virulence-associated genetic polymorphisms of *C. pecorum* [25]. In addition, 6 out of 8 strains from diseased ruminants clustered to a single *ompA* sequence group [25].

In this study we further investigated the *C. pecorum* segregation by virulence in *ompA*, *incA*, and ORF663. These loci were sequenced for an expanded panel of *C. pecorum* isolates from most known hosts of *C. pecorum*, including 11 strains isolated from swine, 24 additional strains isolated from ruminants, and 3 strains isolated from koalas. Virulence associations of *incA* and ORF663 CTRs were confirmed and expanded to porcine and koala *C. pecorum* isolates, and low virulence significantly associated with evolutionary distance of *ompA*, *incA*, and ORF663 from the respective putative *C. pecorum* ancestor.

## Results

### Sequence analysis of *ompA*, *incA* and ORF663

All 32 strains yielded the expected amplification products of *ompA*, *incA*, and ORF663, except for two bovine strains (DC49 and DC55) that failed to give an *incA* and one porcine strain (R106) that failed to give an ORF663 amplicon (Table 1). Sequence analysis of *incA* showed that all 11 porcine strains had one encoded motif (APA) with 7 to 14 repetitions representing 7 variants (Table 1). Similarly, the 3 koala strains with recently deposited genomes showed 4–11 repetitions of the APA motif. In addition, a new motif of 9 nucleotides (GCTGGAGCC) encoding amino acids alanine and glycine AGA (Motif 5) was identified. This motif was detected only in 3 bovine strains isolated from different geographical areas and associated with different conditions (DC13, 2047, 66P130; Table 1).

**Table 1:** Sequence coding tandem repeat characteristics and accession numbers for all *C. pecorum* strains analyzed in this study.

Strain*	*incA* CTR numbers & amino acid motifs	ORF663 15-mer CTRs	*ompA* GenBank #	*incA* GenBank #	ORF663 GenBank #
E58^a^	12 APA	22	EU837071	EU837066	EU837072
LW613* b	9 APA	45	GQ228176	GQ228147	GQ228117
LW623* b	9 APA	45	GQ228177	GQ228148	GQ228118
LW679^c^	11 APA	45	EU684921	EU340821	EU684939
L14* b	11 APA	35	GQ228175	GQ228146	GQ228116
IPA^d^	7 APA	13	AZBD01000001.1	AZBD01000005.1	AZBD01000005.1
FC-Stra* b	7 APA	50	GQ228172	GQ228143	GQ228113
JP1751* b	10 APA	45	GQ228173	GQ228144	GQ228114
SBE^e^	12 APA	22	EU684916	EU340823	EU684934
AKT^f^	8 APA	24	EU684918	EU340816	EU684936
AB10^g^	9 APA	22	EU684917	EU340815	EU684935
VB2^f^	15 APA	24	EU684919	EU340824	EU684937
P787^h^	2 APA+10 APAPE	16	NC_022441.1	NC_022441.1	NC_022441.1
DBDeUG^d^	11 APA	30	AZBB01000001.1	AZBB01000008.1	AZBB01000004.1
L17* i	14 APA	28	GQ228181	GQ228152	GQ228123
PV3056/3^h^	9 APA+1 APE	27	NC_022439.1	NC_022439.1	NC_022439.1
DC13* j	12 AGA	25	GQ228171	GQ228142	GQ228111
iB2^g^	5 APA+3 APEVPA+4 APE	68	EU684925	EU340810	EU684943
iB1^g^	5 APA+3 APEVPA+3 APE	65	EU684924	EU340811	EU684942
C14* j	3 APA+8 APEVPA	46	GQ228169	GQ228140	GQ228109
824^g^	4 APA+8 APEVPA	41	EU684922	EU340809	EU684940
DC49* j	-	23	GQ228195	-	GQ228119
BE53^e^	8 APA	20	EU684923	EU340808	EU684941
L39* i	13 APA	10	GQ228182	GQ228153	GQ228124
L40* i	10 APA	10	GQ228184	GQ228155	GQ228126
L71* b	13 APA	10	GQ228185	GQ228156	GQ228127
HsLuRZ* i	12 APA	10	GQ228183	GQ228154	GQ228125
DC47* j	10 APA	7	GQ228193	GQ228164	GQ228135
L1* i	8 APA	42	GQ228174	GQ228145	GQ228115
R106* i	14 APA	-	GQ228197	GU014536	-
1886* i	10 APA	21	GQ228179	GQ228150	GQ228121
1920BRZ* i	7 APA	42	GQ228168	GQ228139	GQ228108
1710S* b	11 APA	42	GQ228167	GQ228138	GQ228107
1708* b	10 APA	15	GQ228194	GQ228165	GQ228136
29531/1* k	9 APA	5	GQ228189	GQ228160	GQ228131
M14^f^	22 APA	14	EU684920	EU340814	EU684938
5184/4* k	5 APA+3 APEVPA+6 APE	56	GQ228192	GQ228163	GQ228134
DC55* j	-	16	GQ228196	-	GQ228112
DC52* j	12 APA	15	GQ228178	GQ228149	GQ228120
iB3^g^	14 APA	53	EU684926	EU340827	EU684944
MC/MarsBar^d^	4 APA	11	AZBC01000001.1	AZBC01000014.1	AZBC01000008.1
iB4^g^	10 APA+4 APAPE	53	EU684927	EU340826	EU684945
IPTaLE^d^	10 APA	18	AZBE01000002.1	AZBE01000014.1	AZBE01000007.1
R69^l^	2 APA+8 APAPE	58	EU684930	EU340822	EU684948
W73^l^	2 APA+8 APAPE	58	EU684929	EU340825	EU684947
C4* j	3 APA+2 APAVPA	54	GQ228170	GQ228141	GQ228110
3257* k	2 APA	41	GQ228190	GQ228161	GQ228132
66P130* b	10 AGA	21	GQ228180	GQ228151	GQ228122
iB5^g^	12 APA	62	EU684928	EU340817	EU684946
2047* k	12 AGA	16	GQ228191	GQ228162	GQ228133
PV5* k	11 APA	31	GQ228166	GQ228137	GQ228106
748/4* k	3 APA	34	GQ228188	GQ228159	GQ228130
4283/3* k	3 APA	21	GQ228187	GQ228158	GQ228129
3638/3* k	3 APA	21	GQ228186	GQ228157	GQ228128
iC4^g^	6 APA+12 APAPE	60	EU684933	EU340819	EU684951
iC2^g^	6 APA+12 APAPE	52	EU684931	EU340818	EU684949
iC3^g^	6 APA+11 APAPE	59	EU684932	EU340820	EU684950

*Strains sequenced in this study. Strains not marked with an asterisk were sequenced in a preceding study [23], or posted as complete genomes [30]–[32].

a Referenced in [31].

b Referenced in [2].

c Referenced in [42].

d Referenced in [30].

e Isolated by M. Dawson, Virology Department, Central Veterinary Laboratory, Weybridge UK.

f Isolated at INRA, UR1282, Infectiologie Animale et Santé Publique, Centre de Recherche de Tours, France.

g Referenced in [43].

h Referenced in [32].

i Referenced in [3].

j Supplied by Konrad Sachse, Friedrich-Loeffler-Institut Jena, OIE and National Reference Laboratory for Chlamydiosis, 07743 Jena, Germany, 07743 Jena, Germany.

k Supplied by Simone Magnino, Istituto Zooprofilattico Sperimentale della Lombardia e dell’Emilia Romagna “Bruno Ubertini”, National Reference Laboratory for Animal Chlamydioses, Sezione Diagnostica di Pavia, 27100 Pavia, Italy.

l Isolated by M.S. McNulty, Veterinary Research Laboratory, Stormont, Belfast, Ulster.

-not amplified by PCR.

Similar to the ruminant strains isolated from diseased animals, the 10 porcine strains and 3 koala strains, all from diseased hosts, possessed lower numbers of ORF663 CTR repetitions (<43 repeats) than most intestinal strains isolated from asymptomatic (healthy) ruminants (Table 1). Strains isolated from asymptomatic ruminants (n = 19) had more than 43 repeats, except for 6 strains isolated from cattle and one strain isolated from sheep (Table 1).

### Correlation of *ompA* with virulence

**Figure 1:**
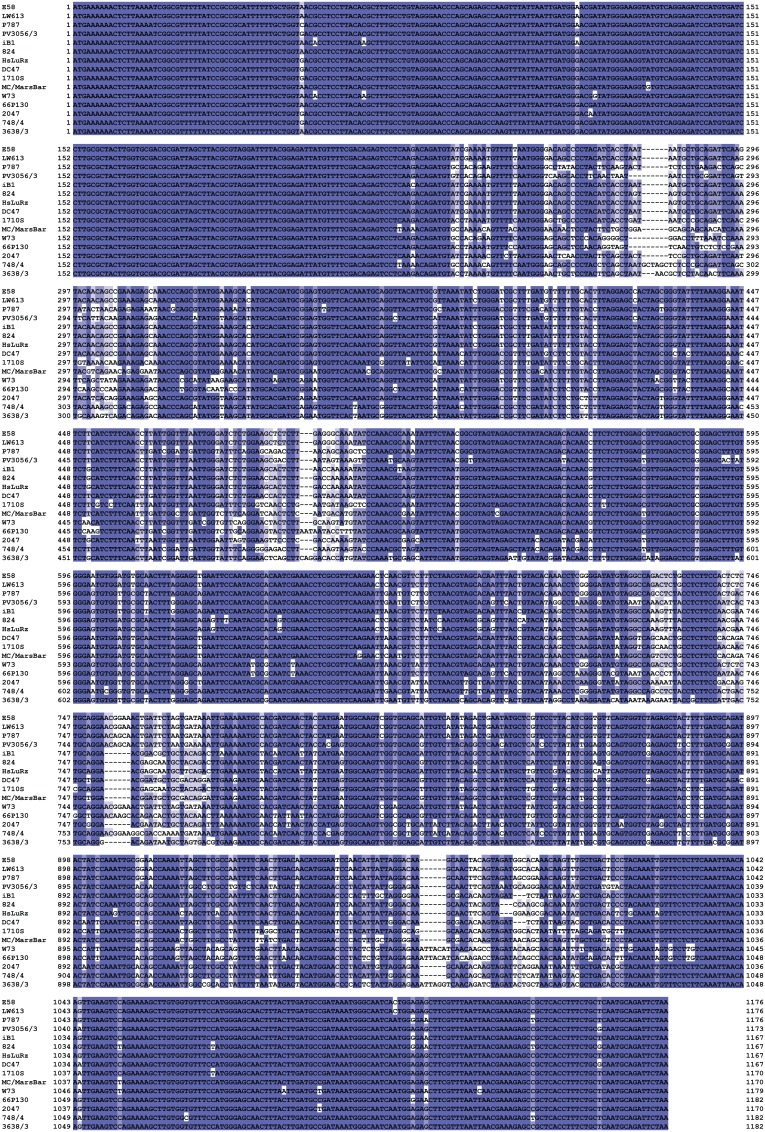
*C. pecorum ompA* alignment. A subset of the 57 analyzed *C. pecorum* strains was selected that represents all major clades of the phylogram in Figure 2. The corresponding sequence alignment of the complete *ompA* of all 57 strains was used to infer *C. pecorum ompA* evolution by construction of a phylogenetic tree. The alignment of the resultant amino acid sequences of all 57 *C. pecorum* OmpA proteins deduced from the nucleotide sequences is shown in Figure S1.

**Figure 2:**
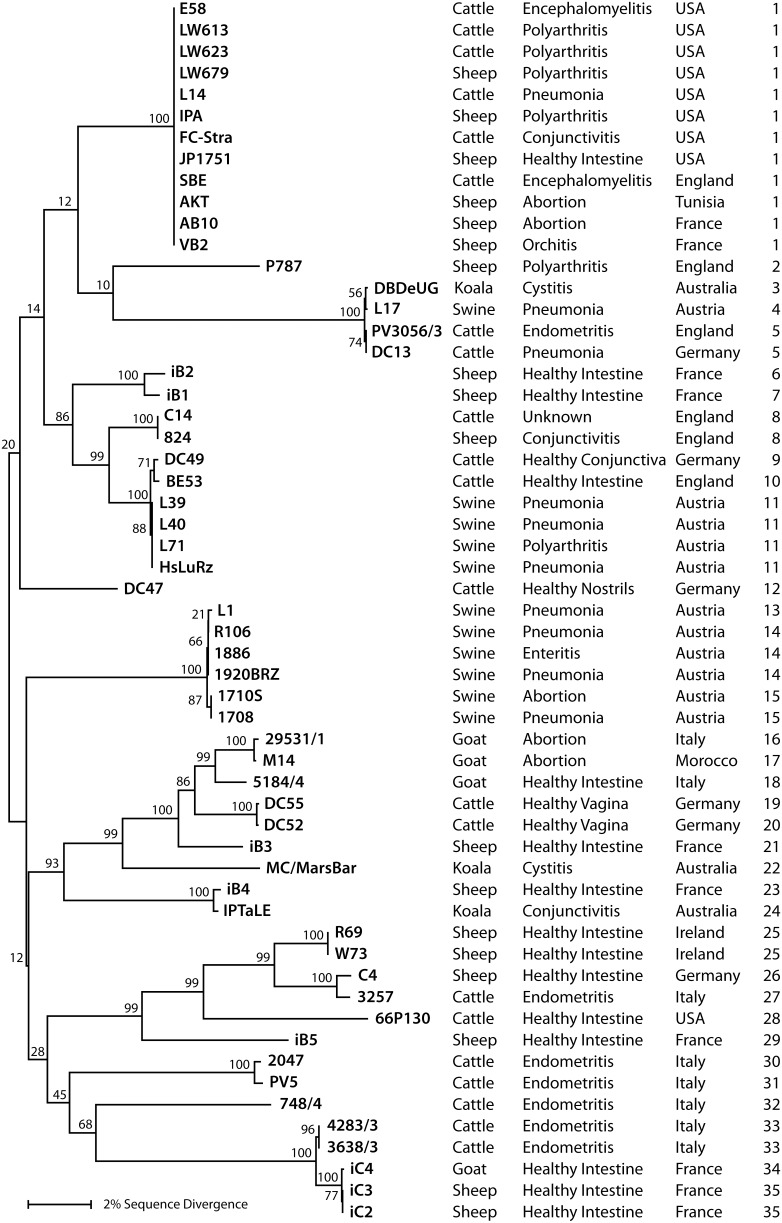
Unrooted neighbor-joining phylogram of *ompA* of 57 *C. pecorum* strains based on the nucleotide sequence alignment. Percentages of branching patterns in bootstrap analyses of the dataset (10,000 replications) are indicated left to the branches. Host animal species, disease association, country of origin, and *ompA* phylogenetic rank are indicated in the columns to the right of the strain names.

The chlamydial *ompA* is one of the most polymorphic of all genes conserved throughout the genus *Chlamydia,* and therefore frequently used as surrogate for approximating overall evolution of chlamydial genomes. This diversity is based not on variation in repeat elements, but on frequent recombination within the 4 variable domains of the gene [25]. We therefore used *ompA* to estimate the correlation between *C. pecorum* genomic diversity with virulence. We first aligned the complete *ompA* sequences of all 57 *C. pecorum* strains used in this study (Figure 1; Figure S1) and performed neighbor-joining phylogenetic reconstruction of their evolution. The resultant phylogram in Figure 2 arranged the *ompA* genes into several clusters of closely related sequences (clades) that were separated from other clades by deep and strongly bootstrap-supported branches [25].

To estimate the association of *ompA* genotype with disease, the deeply separated branches of the *ompA* phylogram in Figure 2 were stratified into 5 high- and 4 low-virulence clades. “High-virulence” clades contained 50% or more strains isolated from diseased animals (strains E58-VB2, P787-DC13, iB2-HsLuRz, L1-1708, 2047-748/4), while “low-virulence” clades contained less than 50% strains isolated from diseased animals (strains DC47, 29531/1-IPTaLE, R69-iB5, 4283/3-iC2). In sum, the high-virulence clades contained 30 high-virulence strains among a total of 35, while the low-virulence groups differed highly significantly and contained 7 high-virulence strains among a total of 21 (*P* = 0.0001; two-tailed Fisher Exact Test).

**Figure 3:**
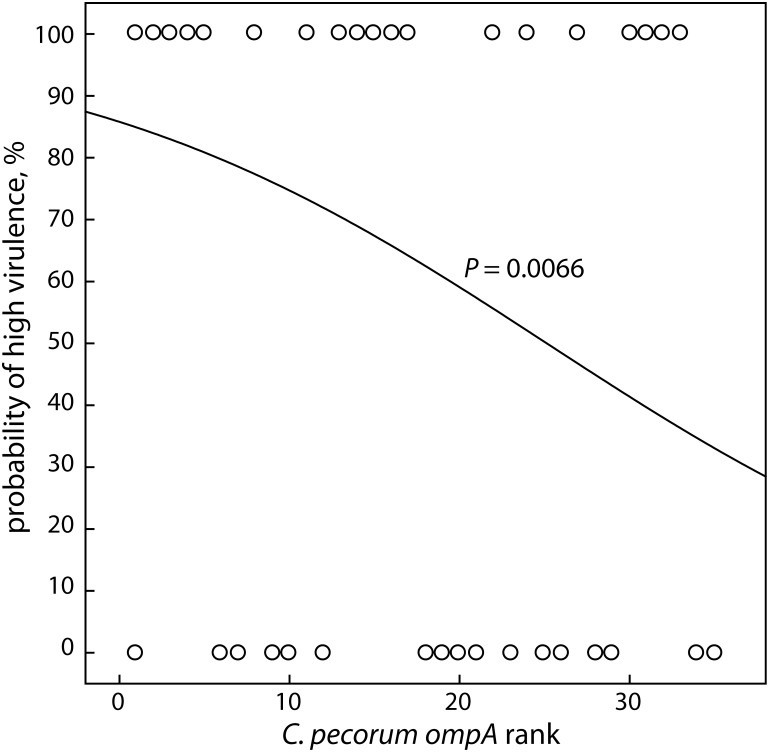
Relationship between virulence of *C. pecorum* strains and rank number in the *ompA* phylogram. The probability of high virulence was determined by logistic regression analysis of the virulence of *C. pecorum* isolates scored by host disease association (0 = healthy; 100 = diseased), and *ompA* rank numbers of the isolates were regressed against virulence. The probability of high virulence decreases highly significantly with increasing *ompA* rank number.

We also used non-stratified *ompA* phylogenetic rank data to evaluate the relation between *ompA* evolution and virulence by logistic regression. All strains with a unique *ompA* genotype received a unique phylogenetic rank number between 1 and 35, based on their position in the phylogram in Figure 2. *C. pecorum* strains isolated from diseased animals were scored as “high virulent” versus the strains isolated from healthy animals scored as “low virulent”. As evident in the highly significant regression plot (*P* = 0.0066; Figure 3), the probability of high virulence was high for low *ompA* rank, but dropped with increasing *ompA* rank. Thus, two analyses indicated that strain position on the *ompA* phylogenetic tree highly significantly correlates with virulence of *C. pecorum* isolates.

### Correlation of *incA*-coding tandem repeat sequence motifs with virulence

Next, we sought to quantitatively assess the relationship between the numbers of repetitions of sequence motifs in *incA* and the virulence of the *C. pecorum* strains. While amino acid APA/AGA motifs are dominant, addition of different motifs (APEVPA, APAVPA, APE, or APAPE) highly significantly associated with low virulence, i.e. 10 of 17 low-virulence strains possessed such sequence motifs, while 30 of 31 high-virulence strains did not (*P*<10^−4^; two-tailed Fisher Exact Test).

**Figure 4:**
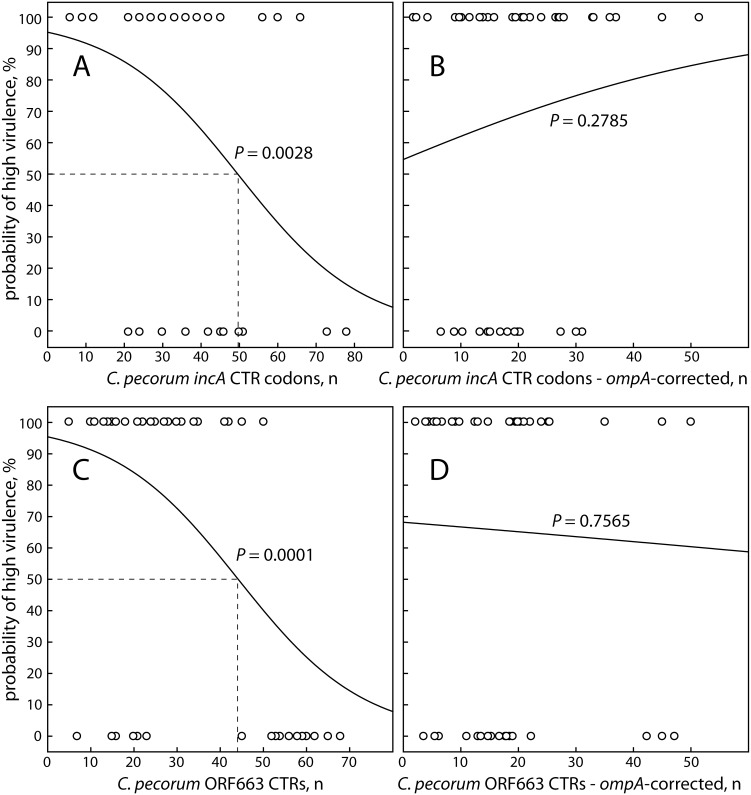
Relationship between virulence of *C. pecorum* strains and coding tandem repeats in *incA* and ORF663. The probability of high virulence was determined by logistic regression analysis of the virulence of *C. pecorum* isolates, scored as in Figure 3, and continuous parameters for CTR numbers of the isolates were regressed against virulence. (A) the number of codons encoded by the CTRs in *incA*; (B) the number of codons encoded by the CTRs in *incA* corrected for the rank number of each isolate in the *ompA* phylogram. (C) The number of CTRs in ORF663; and (D) the number of CTRs in ORF663 corrected for the *ompA* rank number of each isolate. The highly significant correlation between CTRs and probability of high virulence in both *incA* and ORF663 is abolished by correction for *ompA* rank, indicating co-evolution of *ompA*, *incA*, and ORF663.

Similar to the *ompA* phylogenetic rank, we examined the correlation of the number of CTRs in *incA* with virulence by logistic regression. To account for the total amount of the different repeat motif insertions, we used the total number of amino acids encoded by these CTR codons. In logistic regression, high total CTR codon numbers highly significantly correlated with low virulence (*P* = 0.0028), with 50 codons representing a midpoint 50% probability of high virulence (Figure 4A).

A fundamental question in this analysis is whether molecular evolution of the *C. pecorum* strains and *incA* CTR codon numbers is co-linear, and whether, therefore, the correlation between *incA* CTR codons and virulence was confounded by the phylogenetic position of the isolates. To account for *C. pecorum* evolution, we created a corrected *incA* CTR codon dataset that was controlled for the position of the isolates in the *ompA* phylogram. This was achieved by creating standardized phylogram rank data (mean = 0, SD = 1), adjusting these data to positive by adding 1+ the absolute minimum standardized rank number (results in 1 as the minimum adjusted standardized rank number), and dividing the number of *incA* CTR codon of each strain by the respective adjusted standardized *ompA* rank number. Using the *ompA* rank-corrected *incA* CTR codon data, we repeated the logistic regression analysis, but failed to obtain a significant correlation to virulence (*P* = 0.2785; Figure 4B). Thus, both *incA* and *ompA* evolution progress in a co-linear fashion, linking the number of *incA* CTR codons to the phylogenetic position of the *C. pecorum ompA*.

### Correlation of coding tandem repeats in ORF663 with virulence

Similar to *incA*, we examined the correlation of the number of CTRs in ORF663 with virulence. The correlation of the number of CTRs in ORF663 with virulence was highly significant (*P* = 0.0001), again with low numbers of CTRs associating with high probability of high virulence, with 50% probability of high virulence at 43 repetitions (Figure 4C). Interestingly, there is a bimodal distribution of the CTR numbers in low-virulence strains, with 6 bovine strains isolated from healthy animals having less than 24 CTR repetitions, while all other isolates had 43 or more repetitions. When we tested for confounding by phylogenetic position using an *ompA* rank-corrected ORF663 CTR dataset, the correlation was lost (*P* = 0.7565; Figure 4D). Thus, analogous to *incA*, the number of CTRs in *C. pecorum* ORF663 and *ompA* evolution are closely linked.

### Co-evolution of *ompA*, ORF663, and *incA* towards reduced virulence

As a final test for evolutionary linkage, we also tested for co-evolution of the CTR numbers in ORF663 and *incA* by creating an *incA* CTR codon number dataset that was corrected for ORF663 CTR numbers. Again, the ORF663 correction eliminated the correlation between *incA* CTR codons and virulence (*P* = 0.4234; data not shown), thus confirming *incA* and ORF663 co-evolution.

**Figure 5:**
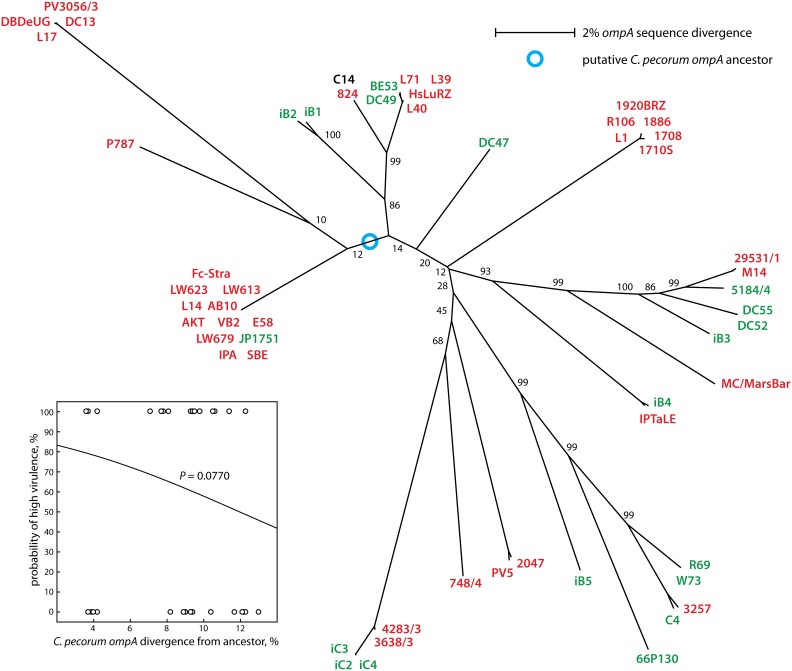
Evolutionary distance from the putative *ompA* ancestor in correlation to virulence of *C. pecorum* strains. A putative ancestral *ompA* was assumed at the connection of an outgroup, composed of one *ompA* sequence each of the 8 remaining chlamydial species, to the 57 *C. pecorum ompA* seqeunces (blue circle). This root is also consistent with an ancestor in the unrooted *ompA* neighbor-joining phylogram (Figure 2) at several poorly resolved and weakly bootstrap-supported branches that link the deep branches of the phylogenetic tree. Bootstrap support is indicated by numbers at branches, but not shown at terminal nodes of deep branches. Branch lengths are proportional to evolutionary *ompA* distance, with the bar indicating 2% sequence divergence (percent nucleotide substitutions). Low-virulence strains are indicated by green font, high-virulence strains by red font. **Inset**: The relationship between *ompA* evolutionary distance from the putative ancestor and the probability of high virulence of *C. pecorum* strains was determined by logistic regression analysis. Long evolutionary distance is correlated to low probability of high virulence, but fails to reach the *P*<0.05 significance threshold.

Collectively, these results suggested that the molecular evolution of *C. pecorum* progressed from ancestral strains with high virulence towards strains with low virulence, and that increased numbers of CTRs in *inc A and* ORF663, as well as recombination in *ompA* resulting in new *C. pecorum* serovars [26], were markers, if not mediators, of this progression towards low virulence. This hypothesis is testable with the present dataset since it implies that phylogenetic sequence divergence of *ompA* from the hypothetical ancestor inversely correlates with virulence of the extant *C. pecorum* strains. We assumed the root of the *ompA* phylogenetic tree at the connection to an outgroup composed of one *ompA* sequence of each of the eight remaining chlamydial species. This putative *C. pecorum* ancestor located to a set of short and weakly bootstrap-supported branches at the base of the phylogram (Figure 5). Association of virulence with evolutionary divergence of each strain from this ancestor was analyzed by logistic regression (Figure 5). While reduced probability of high virulence at long evolutionary distances was obvious from the placement of many strains isolated from healthy animals at the tips of long branches, this trend failed to reach significance (*P* = 0.0770; Figure 5).

**Figure 6:**
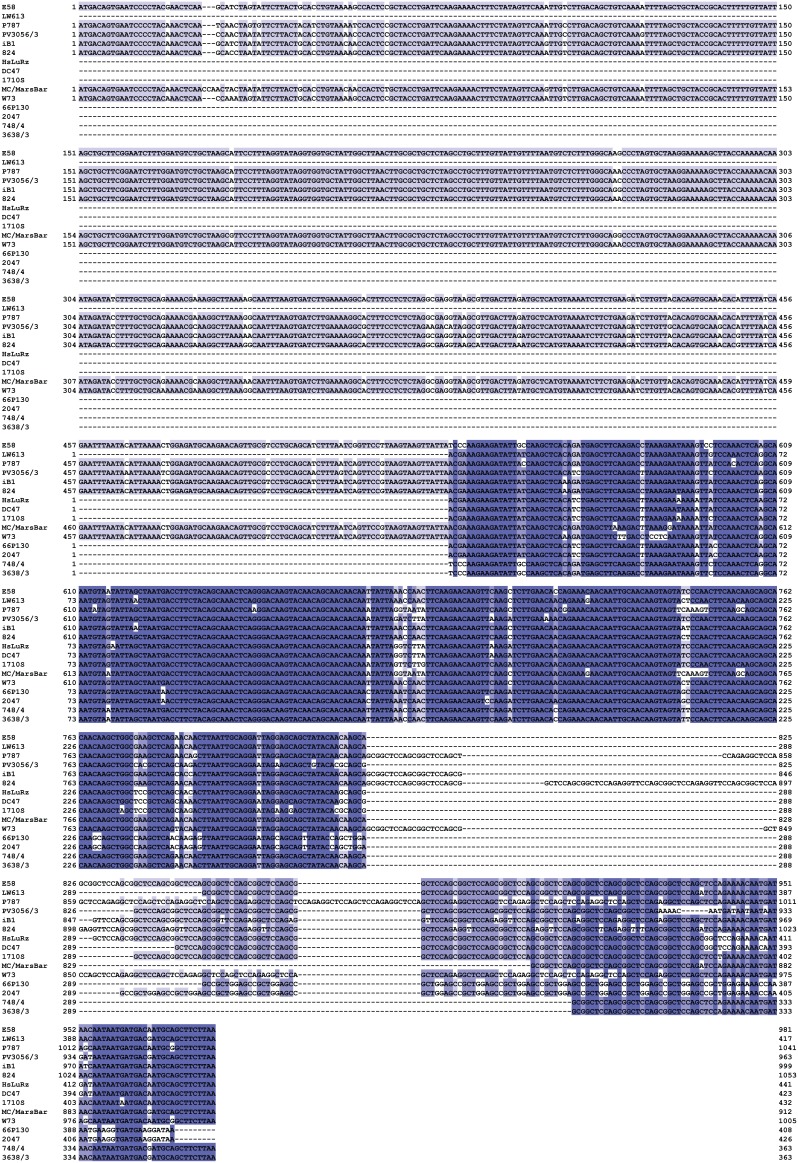
*C. pecorum incA* alignment. The strain subset used in Figure 1 is shown. The complete *incA* is shown for all strains for which the sequence is available, demonstrating highly conserved 5′ (position 1-825 of strain E58) and 3′ ends of the gene (position 906-end of strain E58), interrupted by a highly variable region of coding tandem repeats. The alignment of the PCR fragment sequences available for all 57 strains, corresponding to positions 537 through the 3′ end of strain E58, was optimized for minimal sequence divergence and used for construction of the *C. pecorum incA* phylogenetic tree in Figure 7. The alignment of the resultant amino acid sequences of all 57 partial *C. pecorum* IncA proteins deduced from the nucleotide sequences is shown in Figure S2.

**Figure 7:**
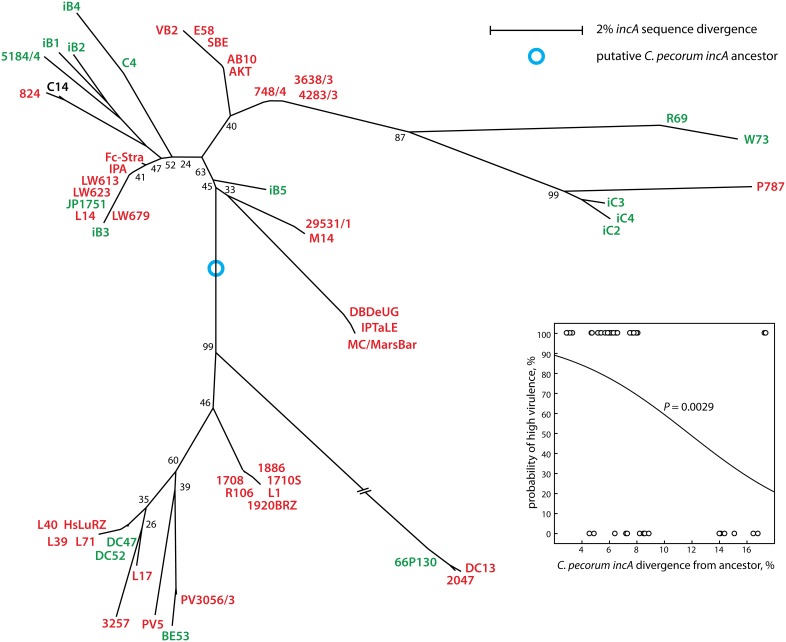
Evolutionary distance from the putative *incA* ancestor correlates to virulence of *C. pecorum* strains. A neighbor-joining phylogram (not shown) was constructed of the conserved 5′ portion of *incA* of all available *C. pecorum* sequences, and an outgroup composed of one *incA* sequence of each of the 8 remaining chlamydial species. In this unrooted phylogram based on the sequence alignment of the 3′ *incA* fragment available for all 57 *C. pecorum* strains in this study, the putative ancestral *incA* was assumed at the connection of this outgroup (blue circle). Indicators of bootstrap support, branch lengths, and strain virulence correspond to Figure 5. **Inset**: The relationship between *incA* evolutionary distance from the putative ancestor and the probability of high virulence of *C. pecorum* strains was determined by logistic regression analysis. Long evolutionary distance is highly significantly correlated to low probability of high virulence.

Next, we examined *incA* for evidence of linkage between virulence and distance from the evolutionary ancestor. Alignment of genes that contain different numbers of CTRs, such as *incA*, is notoriously difficult and very sensitive to the choice of alignment parameters. We optimized the alignment by minimizing average pairwise sequence distance, mainly by setting a high penalty for gap opening, and, less so, for gap extension. The resultant alignment (Figure 6; Figure S2) was used for phylogenetic reconstruction (Figure 7), and the putative ancestor was placed where an outgroup of *incA* homologs from the other eight chlamydial species connected to the phylogram of the highly conserved N-terminal fragment of *C. pecorum incA* (the hypervariable CTR region has no homolog). For *incA*, a highly significant inverse correlation between evolutionary divergence and probability of high virulence was found (*P* = 0.0029; Figure 7).

**Figure 8:**
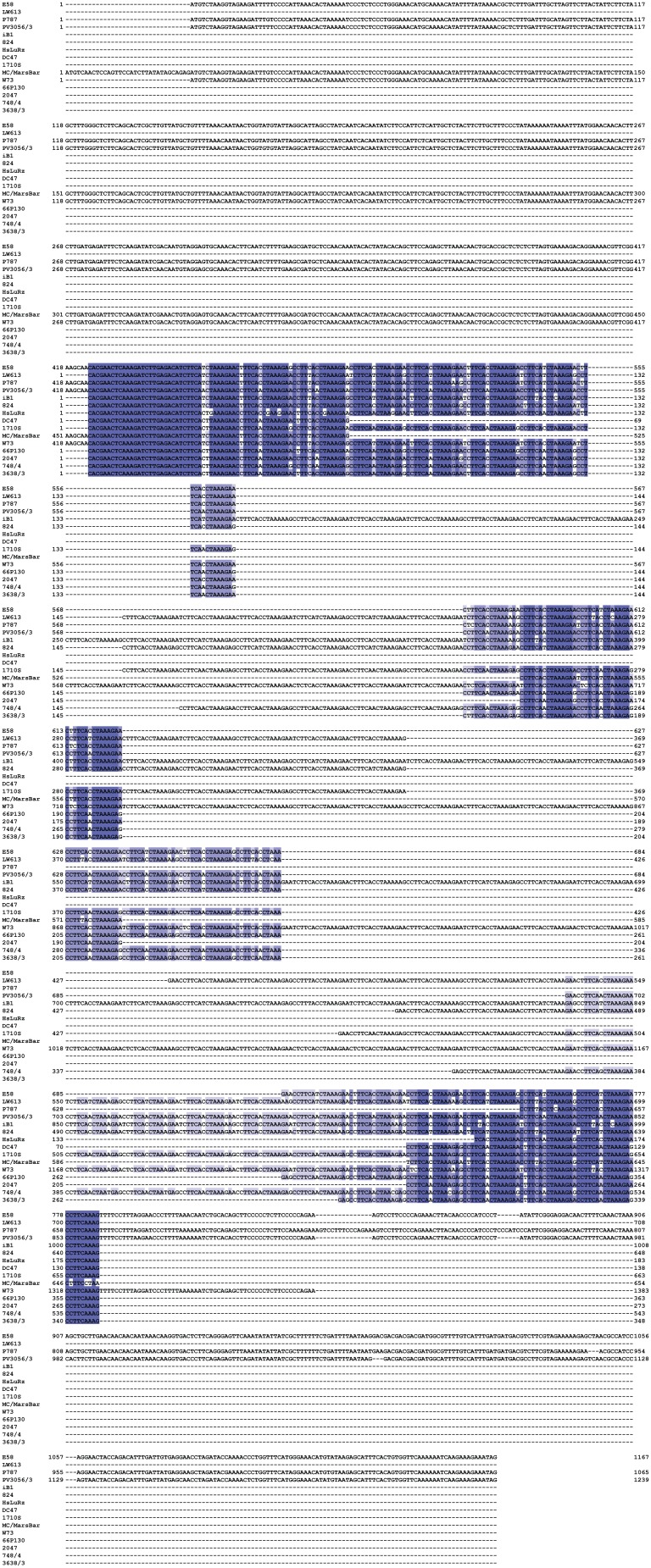
*C. pecorum* ORF663 alignment. The strain subset used in Figure 1 is shown. The complete ORF663 is shown for all strains for which the sequence is available, demonstrating a highly conserved 5′ portion (positions 1–493 of strain E58), followed by a highly variable region of coding tandem repeats containing a short conserved CTR fragment at position 748-786 of strain E58. The alignment of the PCR fragment sequences available for all 57 strains, corresponding to positions 424–786 of strain E58, was optimized for minimal sequence divergence and used for construction of the *C. pecorum* ORF663 phylogenetic tree in Figure 9. The alignment of the resultant amino acid sequences of all 57 full and partial *C. pecorum* IncA proteins deduced from the nucleotide sequences is shown in Figure S3.

**Figure 9:**
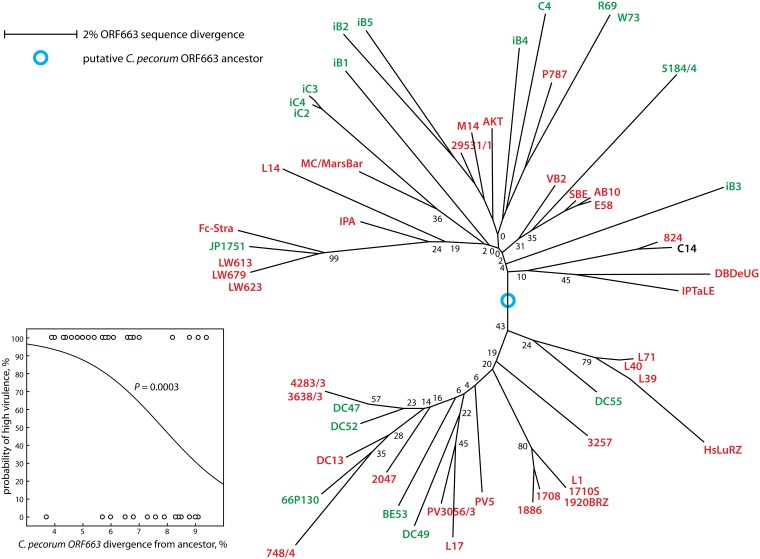
Evolutionary distance from the putative ORF663 ancestor correlates to virulence of *C. pecorum* strains. A neighbor-joining phylogram (not shown) was constructed of the conserved 5′ portion of ORF663 of all available *C. pecorum* sequences, and an outgroup composed of the ORF663 homologs found only in *C. abortus*, *C. psittaci*, C. *caviae*, and *C. pneumoniae*. In this unrooted phylogram based on the sequence alignment of the 3′ ORF663 fragment available for all 57 *C. pecorum* strains in this study, the putative ancestral ORF663 was assumed at the connection of this outgroup (blue circle). Indicators of bootstrap support, branch lengths, and strain virulence correspond to Figure 5. **Inset**: The relationship between ORF663 evolutionary distance from the putative ancestor and the probability of high virulence of *C. pecorum* strains was determined by logistic regression analysis. Long evolutionary distance is highly significantly correlated to low probability of high virulence.

For ORF663, we used an approach similar to *incA* for alignment (Figure 8; Figure S3) and phylogenetic reconstruction (Figure 9). The relationship between long evolutionary distance from the putative ancestor and low virulence was even more pronounced for ORF663 (*P* = 0.0003; Figure 9). Thus, based on phylogenetic modeling, *incA* and ORF663 highly significantly, and *ompA* marginally so, co-evolve towards low virulence, irrespective of the branch of the phylogram, on which a specific strain is located.

### Confirmation of *C. pecorum* gene co-evolution towards low virulence by mean pairwise sequence divergence

If the notion of *C. pecorum* evolution towards low virulence were correct, then a consequence would be that low-virulence strains have travelled a longer evolutionary path than high-virulence strains. This implies that the mean pairwise sequence divergence between low-virulence strains must be higher than that of high-virulence strains, irrespective of the distance from the ancestor, thus providing an easily testable hypothesis. The mean pairwise distances between *ompA*, *incA*, and ORF663 of *C. pecorum* strains isolated from healthy or diseased animals, as well as those of all 57 strains are listed in Table 2. In fact, for all three genes the mean sequence distance between low-virulence isolates from healthy animals is highly significantly by 2–3% higher than that of high-virulence isolates from diseased animals (Table 2). These data provide unambiguous confirmation of *C. pecorum* evolution towards low virulence.

**Table 2:** Mean pairwise sequence divergence between all 57 *C. pecorum* strains analyzed in this study, and between strains isolated from only healthy or from only diseased hosts.

% Pairwise Divergence	Healthy*	Diseased	All
***ompA***	13.23^Ab^ (12.57–13.88)	11.49^C^ (11.10–11.87)	12.49 (12.26–12.72)
***incA***	12.39^Ab^ (11.24–13.55)	9.65^C^ (9.16–10.14)	10.85 (10.51–11.20)
**ORF663**	12.93^AB^ (12.38–13.48)	10.09^C^ (9.81–10.36)	11.13 (10.94–11.31)

*Mean, 95% confidence interval.

A Mean of Diseased highly significantly different at *P*≤10^−5^.

b Mean of All significantly different at *P*<0.05.

B Mean of All significantly different at *P* = 10^−9^.

C Mean of All significantly different at *P*≤10^−4^.

## Discussion

We undertook the present study with the primary aim to identify genetic markers that would allow us to unambiguously discriminate between highly virulent (“pathogenic”) and low-virulence or avirulent (“non-pathogenic”) *C. pecorum* strains that presumably would occupy different branches (clades) of the *C. pecorum* phylogeny. What our results tell us, though, is a different story, in essence that the main driver of reduction in virulence of *C. pecorum* is the distance a strain has traversed in its evolution from the primordial *C. pecorum* strain, and not the phylogenetic position in a specific clade. While it is clear that certain branches of the *C. pecorum ompA* phylogram harbor more highly virulent strains than others, it is uncertain if this is a genetically fixed property of this clade or has more to do with the short evolutionary distance from the ancestor.

The finding of the association of evolutionary distance with virulence is not surprising, given the endemic nature of *C. pecorum* infections in ruminants, swine, and koalas [3], [10], [27], [28], particularly in large herds [29]. Long-term coexistence of host and pathogen results in reduced virulence that is beneficial for the pathogen by maintaining a large host population. Effective adaptation to the host and the self-limiting nature of chlamydial intracellular multiplication may also explain the low number of isolates worldwide despite the ubiquity of *C. pecorum* infections. In addition, we assume that there is a bias towards isolation of *C. pecorum* from diseased animals rather than from healthy ones, because this is what diagnostic laboratories aim for, in particular given the high effort required for isolation of chlamydiae.

In consideration of the potential economic importance of these ubiquitous endemic bacteria [10], [14], [15], we collected a comprehensive set of DNAs of *C. pecorum* strains isolated worldwide from healthy as well as diseased mammalian livestock. Importantly, this study extended previous more limited analyses of ruminant *C. pecorum* strains to include unique sets of *C. pecorum* strains, isolated in Austria from diseased swine [2], [3] and in Australia from diseased koalas [30]. Following a previous investigation, we chose the *ompA*, *incA* and ORF663 loci as targets of our genetic analysis, which have now been identified by genome comparison as being among the most polymorphic genes of the *C. pecorum* genome, which is otherwise more than 99% conserved among the *C. pecorum* strains from which the whole genome is known [30]–[32]. Among these 8 strains, i.e. ruminant *C. pecorum* type strain E58, and strains P787, W73, PV3056/3, and IPA, and koala strains DBDeUG, MC/MarsBar, and IPTaLE, *ompA* is up to 16% divergent, and *incA* and ORF663 up to 8%. This remarkable polymorphism is presumably driven by immunoselection acting on the encoded proteins, all of which have been found immunodominant and eliciting high antibody responses ([3], [33], unpublished data).

The previously identified association of increasing numbers of CTRs in *incA* and ORF663 with reduced virulence [25] was highly significantly confirmed in this study. This finding is in agreement with a study that showed differences between environmental and clinical *Legionella pneumophila* strains in the repeat copy numbers of four genes [34]. Interestingly, six isolates from healthy animals in Germany, England, and the USA, had low numbers of ORF663 CTRs. This indicates that ORF663, as well as *incA* or *ompA*, cannot be used as the sole virulence marker. In *ompA*, specific sequence polymorphisms are not indicators of virulence, however in the context of the overall *C. pecorum ompA* phylogeny they are useful in quantifying distance from the root. As evident in Figure 4, the correlations of all three genes, *ompA*, *incA*, and ORF663 with virulence of the *C. pecorum* isolates are co-linear. Therefore, in combination these 3 genes may serve as probabilistic, but not absolute, markers of virulence.

For the practical use of such molecular markers, their genetic stability under non-selective culture conditions is important, and in fact they remain unchanged in laboratory maintenance of the isolates (data not shown), thus making these genes suitable for highly discriminatory epidemiological studies. At least one of these genes differed for two otherwise identical strains, except for 4 cases, namely LW613 and LW623, 3638/3 and 4283/3, L71 and L39, and E58 and SBE (Figure 2, Table 1), thus uniquely identifying 53 out of 57 *C. pecorum* strains.

The ability of *C. pecorum* to continuously evolve towards low virulence and generate successive allelic variants of *incA*, ORF663, and *ompA* may allow rapid adaptation to a host population and/or evasion of the host immune system. Changes in the repetitive coding regions (loss or gain), mediated by DNA replication error mechanisms, have been shown to cause phase variation in bacteria, which confer major defensive capabilities to the pathogen in order to escape from an aggressive host environment [35], [36]. Similarly, *C. pecorum*, in the process of inserting increasing numbers of CTRs in *incA* and ORF663 and recombining *ompA*, generates new serovars [26] and evolves towards lower virulence. One can speculate that this *ompA* evolution and many CTR insertions in *incA* and ORF663 change the immunological signature of a *C. pecorum* strain. Equally possible, however, is a scenario in which the immunosignature evolution of these genes is accompanied by point mutations in other genes that alter their function, and in that way mediate reduced virulence. Or, alternatively, CTR insertion, as occurs aside from *incA* and ORF663 in multiple other *C. pecorum* proteins such as polymorphic membrane proteins, cytotoxins, and phospholipase D-like proteins [32], may alter both function and immunosignature and mediate virulence reduction by both mechanisms. Therefore, the simultaneous evolutionary changes in *ompA*, *incA*, and ORF663 may or may not be functional correlates of virulence.

A point of criticism of the present analysis of *C. pecorum* virulence may be the fact that the differentiation is based on a single clinical examination of the animal from which the isolate was recovered (Figure 2). The diagnosis in that case may be tenuous in an epidemiological setting with ubiquitous endemic infections of *C. pecorum* [10]. However, the high number of 57 isolates included in this study obtained by numerous investigators over a period of 50 years should alleviate concerns about diagnostic accuracy. If the clinical diagnoses had been widely aberrant, it is very unlikely that we would have been able to demonstrate co-linear correlation with virulence of three independent genetic markers in this study. In addition, Storz *et al*. [37] have experimentally confirmed this genetic differentiation in virulence long ago by experimental oral inoculation of calves, the original host, with *C. pecorum* isolates LW613 or 66P130. Highly virulent strain LW613 caused severe hemorrhagic diarrhea and polyarthritis with predominantly lethal outcome. In contrast, strain 66P130, isolated from feces of a healthy calf, caused only transient mild diarrhea. Thus, experimental inoculation of the original host may produce severe disease only with highly virulent isolates, while such isolates may also be detected in asymptomatic natural infections [15]. These asymptomatic infections, by high- as well as low-virulence *C. pecorum* strains, reduce growth rates in calves by eliciting a status of systemic inflammation [15]. Unraveling the contribution of low- and high-virulence *C. pecorum* strains to performance reduction in livestock will be of great scientific as well as economic interest.

## Methods

### Chlamydial isolates

Thirty two *C. pecorum* strains were newly analyzed in this study, while the remaining 25 isolates had been examined before [24], [25] or published recently [30], [32]. The strains were propagated in the yolk sac of chicken embryos and stored at −70°C as previously described [38]. All isolates were obtained from routine diagnostic specimens in veterinary diagnostics laboratories in Austria (11 isolates), England (1 isolate), Germany (6 isolates), Italy (8 isolates), the USA (6 isolates). The 17 strains from Austria and the USA were isolated between 1965 and 1970, when ethical regulations regarding animal specimens did not exist. The remaining isolates from England, Germany, and Italy were obtained between 1993 and 2006 in governmental veterinary diagnostic laboratories that strictly operated under ethics rules established in the respective countries. None of the specimens obtained caused suffering to the animals in addition to the suffering caused by the natural chlamydial infection.

### PCR conditions and sequencing

PCR was performed according to the GoTaq Flexi DNA Polymerase (Promega, Charbonnieres, France) protocol in a final volume of 50 µL, and consisted of DNA denaturation at 94°C for 5 min, followed by 30 cycles of amplification in a UNO II thermoblock (Biometra, Göttingen, Germany). Each cycle consisted of a denaturation step at 94°C for 30 sec, an annealing step at 55°C (for *ompA* and ORF663) or at 63°C (for *incA*) for 45 sec, an extension step at 72°C for 1 min, followed by a final chain elongation at 72°C for 7 min. The primer pairs used in this study except for forward *incA* primer b15-F (5′-CAAGAACAGTTGCGTCCTG-3′) have been described before [25]. The PCR products were sequenced by automated sequencing (Genome Express, Meylan, France). The complete DNA sequences of *ompA* genes and partial sequences of *incA* and ORF663 genes were deposited in GenBank under accession numbers listed in Table 1.

### Sequence alignment and analysis

The number of repetitions of 15-mer CTR in ORF663 was identified using Tandem repeat finder software [39]. Deduced amino acid sequences were first aligned in the freeware MEGA6 [40] by use of the MUSCLE algorithm that considered for the nucleotide alignment all codon positions according to the Blosum 62 AA substitution matrix. Two obvious sequencing errors in the IPTaLE *incA* between positions 354–367 were manually corrected. Evolutionary distances were computed in MEGA6 in a maximum composite likelihood model as the number of base substitutions per site. Alignments were optimized by varying alignment parameters, in particular gap opening and extension penalties. A gap opening penalty of −5 and extension penalty of −1 resulted in minimum average pairwise sequence distances for all 3 genes, and was used to construct sequence alignments. Publication quality alignments were produced in freeware toolkit Jalview [41]. The evolutionary history was inferred by phylogenetic reconstruction by the neighbor-joining method in the freeware MEGA6 [40], with gaps removed from calculation by pairwise deletion.

### Statistical analysis

Virulence association of *ompA* phylogram clades of and novel *incA* repeat sequences was analyzed by two-tailed Fisher Exact test. Correlation of *C. pecorum* strain virulence with *ompA* rank, *incA* CTR codon numbers, CTRs in ORF663, and the *ompA*, *incA*, and ORF663 nucleotide sequence divergence from the respective ancestral *C. pecorum* gene was determined by logistic regression analysis. Differences in mean pairwise sequence divergence were analysed by Student’s t-test. All statistical analyses were performed by use of the Statistica 7.1 software package (Statsoft, Tulsa, Oklahoma, USA).

## Supporting Information

Figure S1 ***C. pecorum*** **OmpA protein alignment.** Full-length peptide sequences of all 57 analyzed *C. pecorum* strains are shown. The background colors follow the Zappo color scheme for visualization of multi-peptide alignments [41], and correspond to alignment quality determined by amino acid identities and physicochemical similarities according to the Blosum 62 matrix (Pink = aliphatic/hydrophobic aa I, L, V, A, M; orange = aromatic aa F, W, Y; blue = positive aa K, R, H; red = negative aa D, E; green = hydrophilic aa S, T, N, Q; purple = conformationally special aa P, G; yellow = C). Four variable domains, distinguished by the gap insertions in the alignment, are interspersed between 5 highly conserved domains of the OmpA protein.
(TIF)

Figure S2 ***C. pecorum*** **IncA protein alignment.** IncA peptide sequences of all 57 analyzed *C. pecorum* strains are shown. The complete IncA protein was used for alignment, and all available full-length sequences are shown in addition to the sequences encoded by the PCR fragment available for all strains. Amino acids 180 through C-terminal amino acid 326 of strain E58 correspond to the PCR fragment sequence used for phylogenetic reconstruction. Background Zappo colors correspond to alignment quality according to the Blosum 62 matrix. A highly conserved N-terminal region of approximately 275 amino acids is followed by a hypervariable region of inserted coding tandem repeats followed by a short conserved C-terminus of the IncA protein.
(TIF)

Figure S3 ***C. pecorum*** **ORF663 protein alignment.** ORF663 peptide sequences of all 57 analyzed *C. pecorum* strains are shown. The complete ORF5663 protein was used for alignment, and all available full-length sequences are shown in addition to the sequences encoded by the PCR fragment available for all strains. Amino acids 142-262 of strain E58 correspond to the PCR fragment sequence used for phylogenetic reconstruction. Background Zappo colors correspond to alignment quality according to the Blosum 62 matrix. A highly conserved N-terminal region of 154 or 165 amino acids is followed by a hypervariable region of inserted coding tandem repeats followed by short or long variants of a conserved C-terminus of the ORF663 protein.
(TIF)
