# Supplementary figures and images for: Host Adaptation of Chlamydia pecorum towards Low Virulence Evident in Co-Evolution of the ompA, incA, and ORF663 Loci
Source: PLoS One. 2014 Aug 1;9(8):e103615. doi: 10.1371/journal.pone.0103615 (PMC4118914; doi:10.1371/journal.pone.0103615)

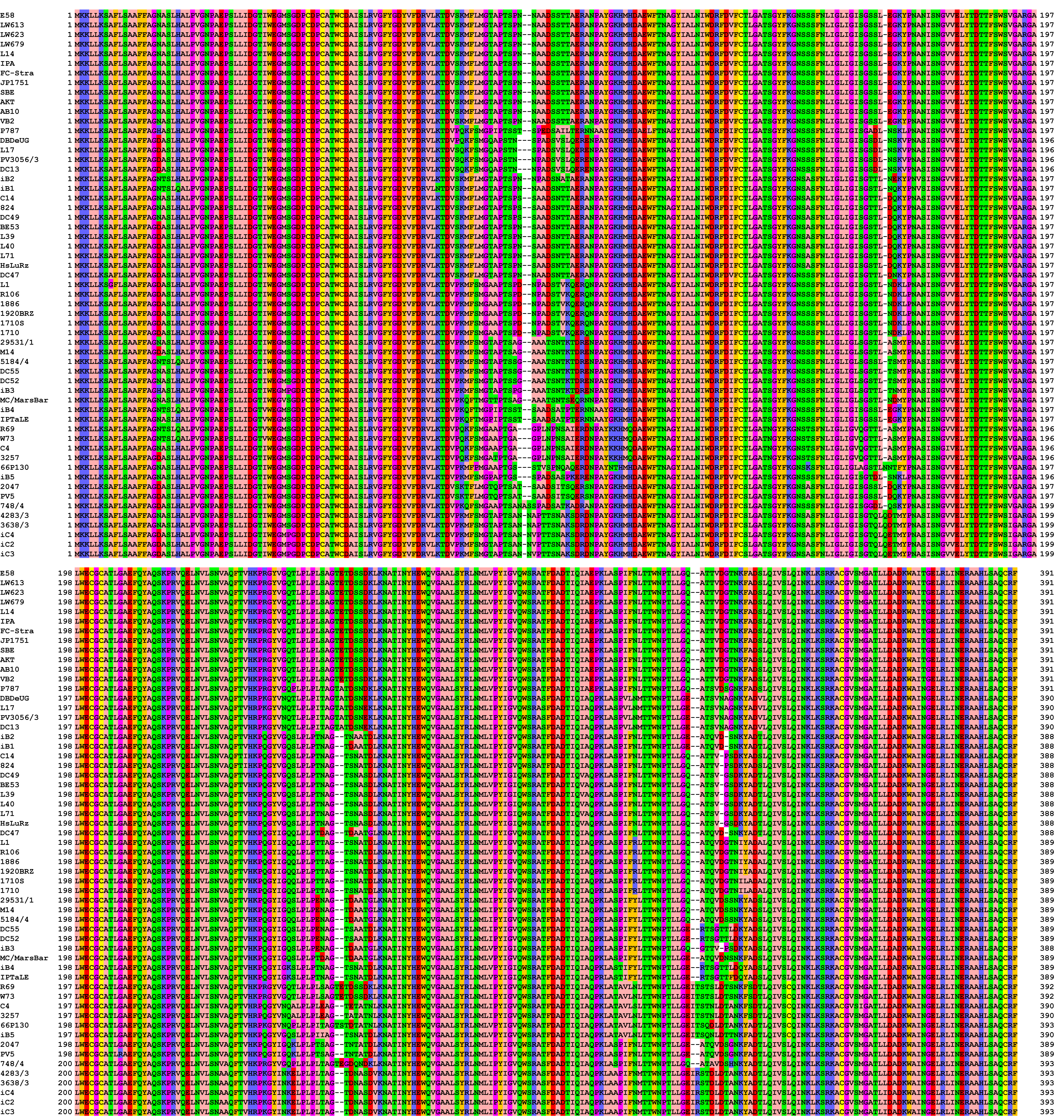

Supplement: Figure S1 — C. pecorum OmpA protein alignment. Full-length peptide sequences of all 57 analyzed C. pecorum strains are shown. The background colors follow the Zappo color scheme for visualization of multi-peptide alignments [41], and correspond to alignment quality determined by amino acid identities and physicochemical similarities according to the Blosum 62 matrix (Pink = aliphatic/hydrophobic aa I, L, V, A, M; orange = aromatic aa F, W, Y; blue = positive aa K, R, H; red = negative aa D, E; green = hydrophilic aa S, T, N, Q; purple = conformationally special aa P, G; yellow = C). Four variable domains, distinguished by the gap insertions in the alignment, are interspersed between 5 highly conserved domains of the OmpA protein. (TIF) [file pone.0103615.s001.tif]

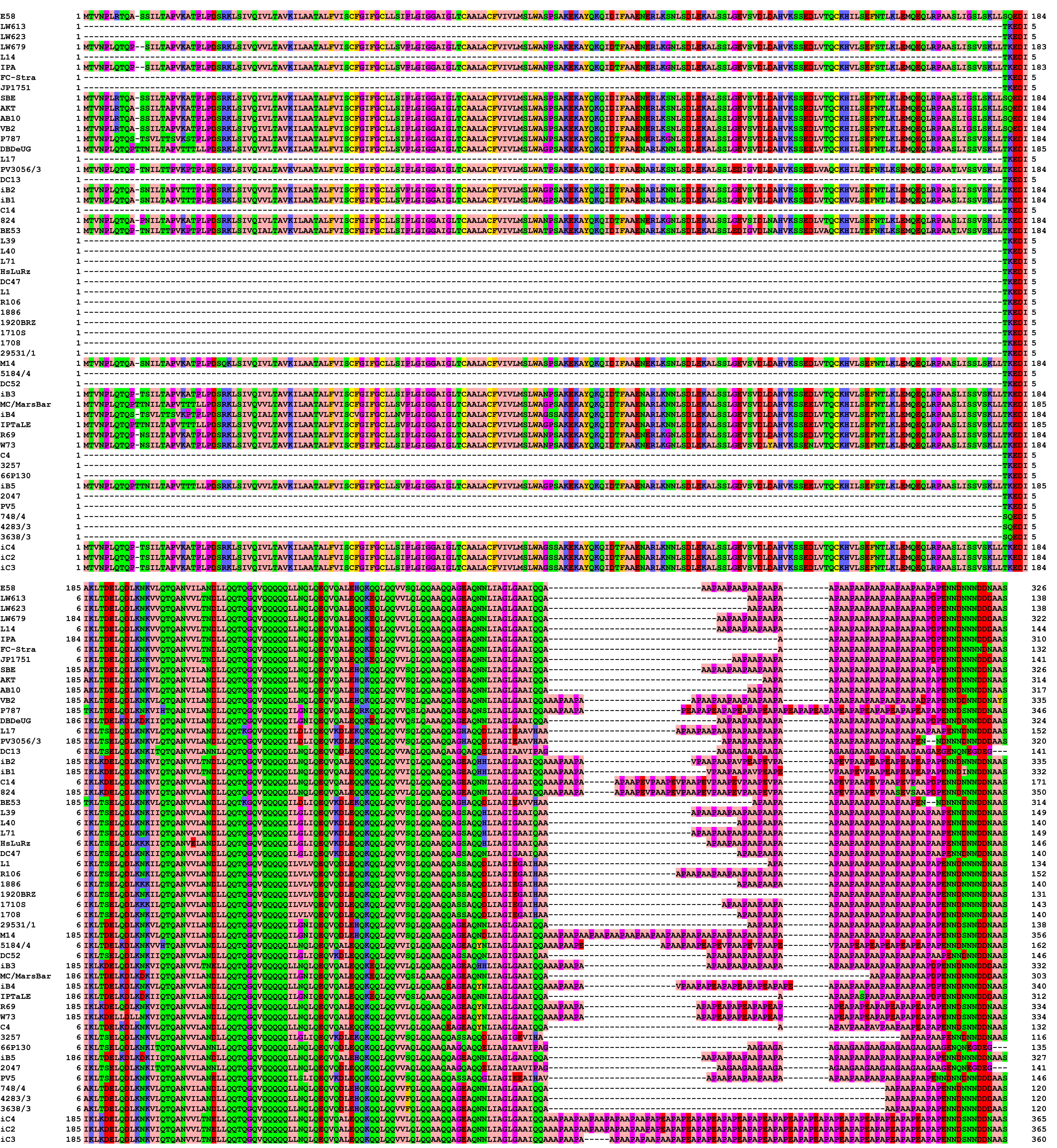

Supplement: Figure S2 — C. pecorum IncA protein alignment. IncA peptide sequences of all 57 analyzed C. pecorum strains are shown. The complete IncA protein was used for alignment, and all available full-length sequences are shown in addition to the sequences encoded by the PCR fragment available for all strains. Amino acids 180 through C-terminal amino acid 326 of strain E58 correspond to the PCR fragment sequence used for phylogenetic reconstruction. Background Zappo colors correspond to alignment quality according to the Blosum 62 matrix. A highly conserved N-terminal region of approximately 275 amino acids is followed by a hypervariable region of inserted coding tandem repeats followed by a short conserved C-terminus of the IncA protein. (TIF) [file pone.0103615.s002.tif]

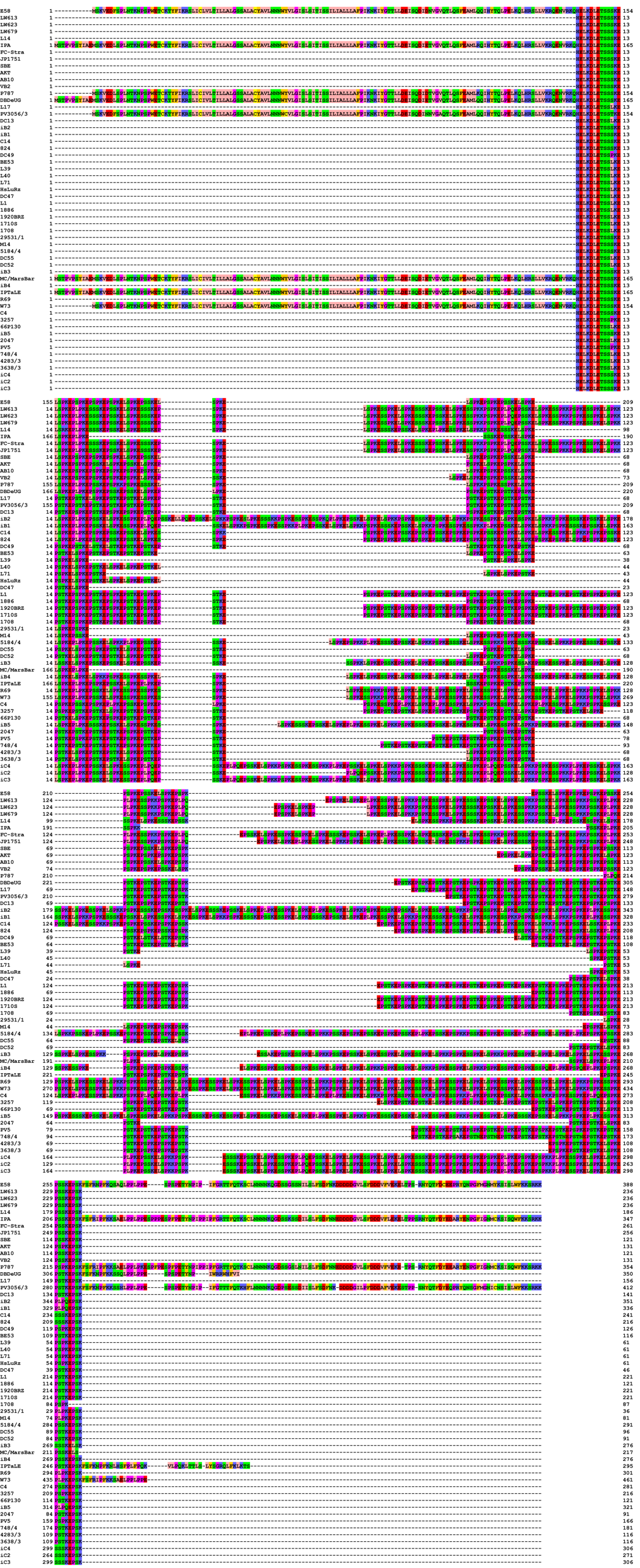

Supplement: Figure S3 — C. pecorum ORF663 protein alignment. ORF663 peptide sequences of all 57 analyzed C. pecorum strains are shown. The complete ORF5663 protein was used for alignment, and all available full-length sequences are shown in addition to the sequences encoded by the PCR fragment available for all strains. Amino acids 142-262 of strain E58 correspond to the PCR fragment sequence used for phylogenetic reconstruction. Background Zappo colors correspond to alignment quality according to the Blosum 62 matrix. A highly conserved N-terminal region of 154 or 165 amino acids is followed by a hypervariable region of inserted coding tandem repeats followed by short or long variants of a conserved C-terminus of the ORF663 protein. (TIF) [file pone.0103615.s003.tif]
